# The effect of screening doors and windows on indoor density of *Anopheles arabiensis* in south-west Ethiopia: a randomized trial

**DOI:** 10.1186/1475-2875-12-319

**Published:** 2013-09-12

**Authors:** Fekadu Massebo, Bernt Lindtjørn

**Affiliations:** 1 Department of Biology, Arba Minch University, Arba Minch, Ethiopia; 2 Centre for International Health, University of Bergen, Bergen, Norway

**Keywords:** *Anopheles arabiensis*, Screening doors and windows, Indoor density, Metal mesh

## Abstract

**Background:**

Screening of houses might have impact on density of indoor host-seeking *Anopheles* mosquitoes. A randomized trial of screening windows and doors with metal mesh, and closing openings on eves and walls by mud was conducted to assess if reduce indoor densities of biting mosquitoes.

**Methods:**

Mosquitoes were collected in forty houses using Centers for Diseases Control and Prevention (CDC) light traps biweekly in March and April 2011. A randomization of houses into control and intervention groups was done based on the baseline data. Windows and doors of 20 houses were screened by metal mesh, and openings on the walls and eves closed by mud and the rest 20 houses were used as control group. Mosquitoes were collected biweekly in October and November 2011 from both control and intervention houses. A Generalized Estimating Equations (GEE) with a negative binomial error distribution was used to account for over dispersion of *Anopheles arabiensis* and culicine counts and repeated catches made in the same house.

**Results:**

Screening doors and windows, and closing openings on eves and wall by mud reduced the overall indoor densities of *An. arabiensis* by 40%. The effect of screenings pronounced on unfed *An. arabiensis* by resulting 42% reduction in houses with interventions. The total costs for screening windows and doors, and to close openings on the eves and walls by mud was 7.34 USD per house.

**Conclusion:**

Screening houses reduced indoor density of *An. arabiensis,* and it was cheap and can easily incorporated into malaria vector strategies by local communities, but improving doors and windows fitness for screening should be considered during house construction to increase the efficacy of screenings.

## Background

Malaria vectors control depends mainly on personal protection, environmental management and use of insecticides for indoor residual spraying (IRS) and mosquito net treatment. The efficacy of long-lasting insecticidal nets (LLITNs) and IRS was reduced in an area where malaria vectors were resistant to insecticide in Benin [1]. In Ethiopia, resistance to pyrethroid insecticides by *Anopheles arabiensis* is increasing [2-4] and, hence, integrated malaria vectors control approach is needed to reduce the challenge from resistance on malaria transmission [5].

Mosquito-proofing houses have a historical success against malaria vectors [6,7]. In Missouri, USA, screened houses afforded a considerable degree of protection against malaria vectors and the incidence of malaria was higher in houses without screening where the population was most accessible for biting mosquitoes [8]. Similarly, in Tennessee River area in USA a substantial reduction of the incidence of malaria was obtained by improving rural houses [7]. Recently, modification of houses reduced houses entry of *Anopheles gambiae* by 78% to 80% in The Gambia [9]. Forty three percent reduction of house entry of *An. gambiae* was reported by closing eves of houses [10]. Screening houses using mosquito proofing materials significantly reduced indoor density of host seeking *An. gambiae*[6,11], and it provides equal protection for all occupants in the houses against bites of malaria vectors [12]. *Anopheles arabiensis* predominantly bites humans indoors in study site [4], hence there is a need for additional malaria vector control to reduce house entry and minimize indoors human-vector contact, and divert them to non-human hosts available outdoors. The objective of this study was to assess whether screening windows and doors by metal mesh, and closing openings on eves and walls by mud would reduce indoor densities of *An. arabiensis* in south-west Ethiopia*.*

## Methods

### Trial design

A randomized control trial was conducted to assess the efficacy of screening windows and doors with metal mesh, and closing openings on eves and walls by mud on indoor density of *An. arabiensis*. The study was done in Chano, a village 15 km north of Arba Minch town in southwest Ethiopia. The nearest sub-village to Lake Abaya (1,350 to 1,850 m from the shore of Lake Abaya, the major larval breeding sites) was purposely selected for screening trial because both epidemiological [13] and entomological [4,14] findings have shown higher risk malaria exposure in this sub-village than other sub-villages. The detail description of the study area has been reported elsewhere [4,14].

### Participants

Forty houses with thatched roof, similar size, found between 1,350 -1,570 m from the main mosquito breeding sites (shore of Lake Abaya), with the number of occupants greater or equal to four and with same number of doors and windows were included for the trial.

### Pre-screening mosquito collections

Mosquitoes were collected from all the 40 houses every second week in four consecutive nights per week (10 CDC light traps per night) in April and May 2011. A total of 160 Centers for Diseases Control and Prevention (CDC) light trap nights were conducted to generate the baseline data. Anophelines were identified using a morphological key [15] and classified into unfed, freshly fed, half gravid and gravid based on abdominal condition. Culicines were counted and discarded.

### Randomization

**Figure 1:**
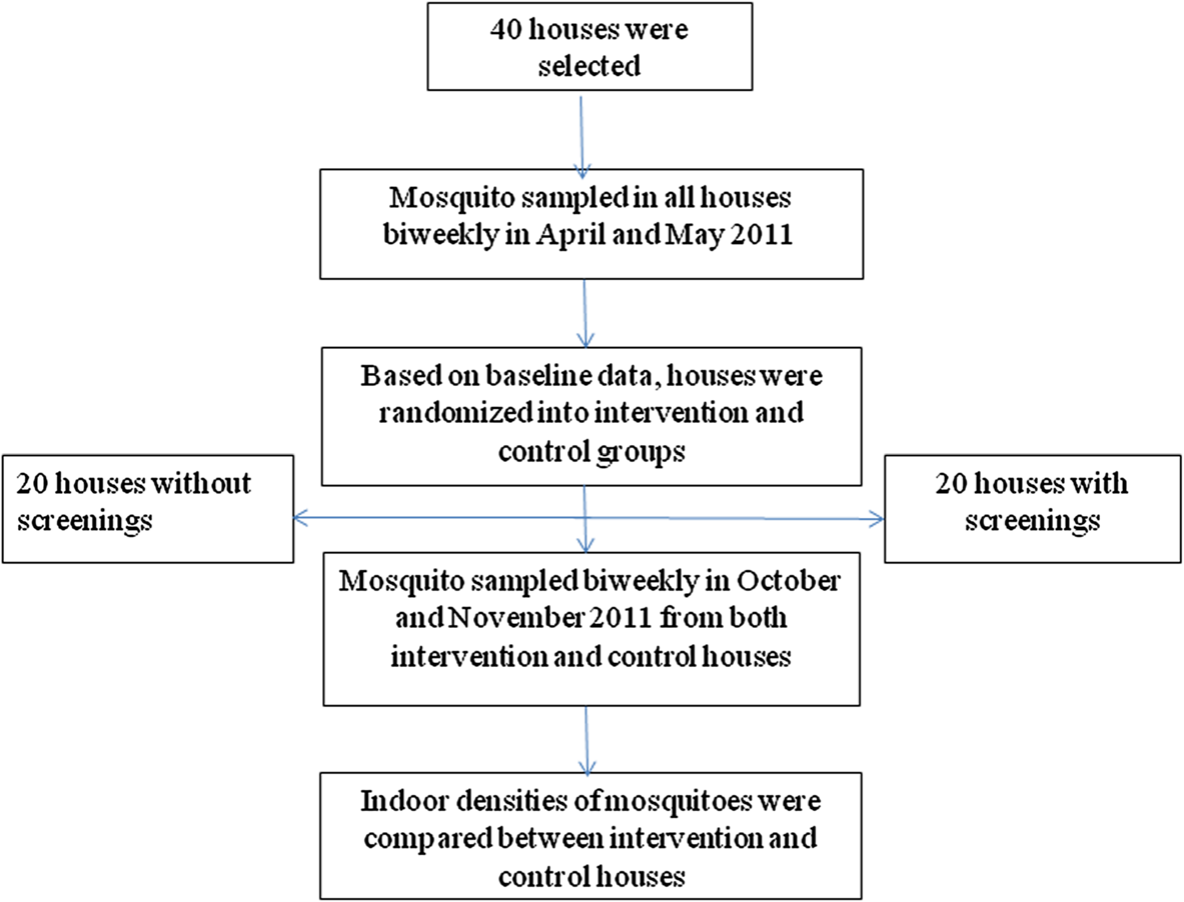
Study design.

Based on the baseline data, the 40 houses were simply randomized into control and intervention groups using IBM SPSS version 20 (Figure 1). The unit of randomization was an individual house. Table 1 shows the baseline data on number of *An. arabiensis* per CDC light trap per night of the two groups which were similar.

**Table 1:** **The baseline data of the mean number of *An. arabiensis *per CDC light trap night (April and May 2011)**

**Abdominal condition**	**Pre-control houses (n = 20)**	**# (95%CI)**	**Pre-intervention houses (n = 20)**	**# (95%CI)**
	**N (no. of mosquitoes)**		**N (no. of mosquitoes)**	
Unfed	683	8.5 (2.3, 14.7)	624	7.8 (4.8, 10.8)
Fresh fed	580	7.2 (4.1, 10.4)	639	7.9 (4.4, 11.5)
Half gravid	105	1.3 (0.7, 1.9)	93	1.2 (0.7, 1.7)
Gravid	240	3 (1.8, 4.2)	269	3.3 (2, 4.7)
Overall	1608	20.1(10.9, 29.3)	1625	20.3 (12.8, 27.8)

#Mean number of *An. arabiensis* per CDC light trap per night; Pre-control houses = houses randomized as control group during intervention; Pre-intervention houses = houses randomized for screening during intervention.

### Interventions

**Figure 2:**
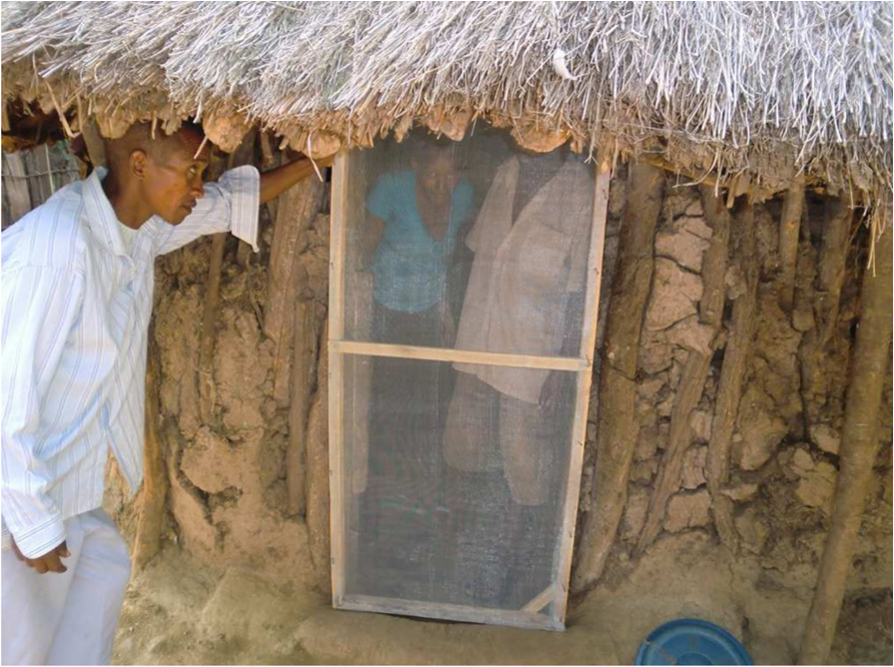
External view of screened door.

**Figure 3:**
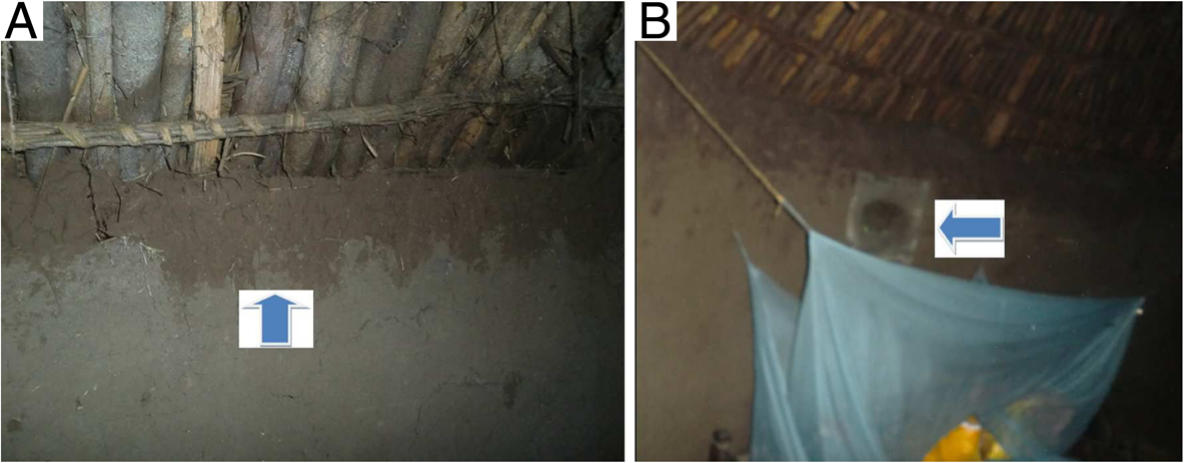
Openings around eves closed by mud (A) and openings for ventilation closed by metal mesh (B).

Doors and windows of the 20 houses were screened by metal mesh (Figure 2), and openings in the walls and eves were closed with mud (Figure 3) to see if screening the doors and windows reduce house entry and indoor density of host seeking *An. arabiensis*. Any openings in the wall for ventilation purpose were closed by metal mesh only. Timber-frame was used for screening doors. The screened doors were fixed on the frame of the main door externally using hinges, and were removed by rolling to enter or leave the houses*.* Windows were permanently fixed externally by metal mesh after getting permission from house owners. The costs for metal mesh, timber frame, nails and labour were calculated.

### Post-screening mosquito collections

The 40 houses were sampled every second week in October and November 2011 by taking five houses from intervention group and five houses from control group per night for four consecutive nights per week. Anophelines were identified using a morphological key [15] and classified into unfed, freshly fed, half gravid and gravid based on abdominal condition. Culicines were counted and discarded.

### Outcome variable

The outcome variable of this study was indoor densities of *An. arabiensis* collected per CDC light trap per night. Mosquito collectors were not masked because CDC light traps are not depending on human skills.

### Statistical analysis

Mosquito data within household was described by mean number of *An. arabiensis* per CDC light trap per night. A Generalized Estimating Equations (GEE) with a negative binomial error distribution was used to account for over dispersion of *An. arabiensis* and culicine counts. A first-order autoregressive correlation structure was considered to account a serial correlation between repeated catches made in the same house. The GEE was fitted separately to counts of different abdominal conditions of *An. arabiensis* and overall culicine to determine the protective effect of screenings against house entry of the species. The mean’s ratio of mosquitoes between screened and control houses were used to determine the percentage reduction of house entry. Non-parametric correlation was used to see the house entry patterns of *An. arabiensis* in pre-intervention and post-intervention months. All houses were included in analysis because no damaged metal mesh and malfunctioned CDC light traps were observed. The statistical significance of screening effect was tested by P-value obtained from GEEs at 0.05 level. IBM SPSS version 20 (SPSS Inc, Chicago, USA) was used for data entry and analysis.

### Ethical conditions

A verbal consent was obtained from the household head and they provided with insecticide untreated bed nets.

## Results

### Mosquito abundance and species composition

A total of 4,778 anophelines and 3,111 culicines were collected during the study period. *Anopheles arabiensis* was the predominant (n = 4249, 89%) species followed by *Anopheles marshalli* (n = 246, 5.1%) and *Anopheles pharoensis* (n = 178, 3.7%). *Anopheles demeilloni, Anopheles dancalicus, Anopheles cinctus, Anopheles culicifacies, Anopheles funestus, Anopheles obscures, Anopheles tenebrosus, Anopheles parensis, Anopheles rufipes, Anopheles ziemanni, Anopheles garnhami* and *Anopheles salbaii* accounted only 2.2% (n = 105).

### House entry patterns of *Anopheles arabiensis* at different months

House entry of *An. arabiensis* followed similar patterns before and during intervention. Households with a maximum number of *An. arabiensis* in the months prior to intervention received higher number during intervention both in control houses (r = 0.72, p <0.001) and houses that were subsequently screenings (r = 0.56, p = 0.01).

### The efficacy of intervention on indoor density of *An. arabiensis*

The efficacy of screening doors and windows on indoor density of *An. arabiensis* is shown in Table 2. The mean number of *An. arabiensis* was 7.9 (95% Wald Confidence Interval (CI): 6.5, 10.1) per CDC light trap per night in non-screened houses, compared with 4.8 (95% Wald CI: 3.9, 6.2) per CDC light trap per night in houses with screens. There was 40% fewer *An. arabiensis* in houses with interventions than those without interventions (ratio of means 0.6, p = 0.006). The indoor density of hunger *An. arabiensis* was reduced by 42% in intervention group (ratio of means 0.58, p = 0.004). The intervention also had an impact on indoor density of freshly fed *An. arabiensis* by resulting 36% reduction of house entry.

**Table 2:** **The efficacy of doors and windows screening on indoor host seeking densities of *An. arabiensis *(October and November 2011)**

**Abdominal condition**	**Control N**	**# (Wald 95%CI)**	**Intervention N**	**#(Wald 95%CI)**	**Means ratio**	**% reduction**	***p***
Unfed	189	2.4 (2.2, 2.7)	115	1.4 (1.1, 1.9)	0.58	42	0.004
Fresh fed	227	2.8 (2.3, 3.6)	143	1.8 (1.5, 2.1)	0.64	36	0.001
Half gravid	13	0.15 (0.1, 0.4)	10	0.13 (0.1, 0.3)	0.87	13	0.83
Gravid	197	2.5 (1.9, 3.5)	122	1.5 (1.2, 1.9)	0.60	40	0.002
Overall	626	7.9 (6.5, 10.1)	390	4.8 (3.9, 6.2)	0.60	40	0.006

# Mean number of *An. arabiensis* per CDC light traps per night.

**Figure 4:**
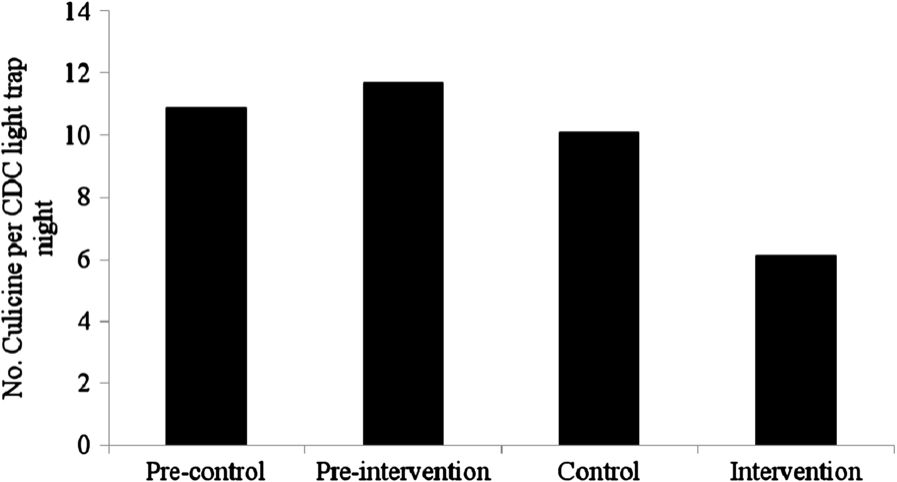
The efficacy of intervention on indoor density of culicine mosquitoes from Chano in southwest Ethiopia.

Figure 4 shows the baseline data and the efficacy of intervention against culicine mosquitoes. The mean number of culicine mosquitoes was 10.1 (95% Wald CI: 8.8, 11.9) in houses without interventions and 6.1 (95% Wald CI: 5, 7.8) in screened houses resulting a 40% reduction in door density of biting nuisance culicine mosquitoes. The total costs for screening windows and doors, and to close openings on the eves and walls by mud was 7.34 USD per household (Table 3).

**Table 3:** Costs for intervention

**Materials**	**Cost per household**
Cost for metal mesh	1.44 USD * 2.5 m = 3.6 USD
Cost for screening including nails and wooden materials	2.3 USD
Closing openings with mud	1.44 USD
Total cost per house	7.34 USD

Total number of houses = 20.

## Discussion

The results of this randomized trial show that screening doors and windows, and closing openings on walls and eves by mud reduced the overall indoor densities of *An. arabiensis* by 40%. Although screening intervention reduced indoor density of *An. arabiensis* at all abdominal stages, the reduction was substantially higher against unfed *An. arabiensis*. The intervention was based on locally bought materials, and was affordable.

The houses we assessed were grass thatched, and doors and windows were not well-suited for screenings. The incompatible of doors for screening might reduce the efficacy in such house types. The roofs of grass thatched local houses prevent opening of screened doors outward; consequently, the screened doors were not permanently fixed and people might not used them constantly during the nights before collection.

A house screening study from The Gambia resulted in 43% reduction of house entry of *An. gambiae* which is comparable to the current study [10]. Although the incidence of malaria infection was not assessed, the previous studies have shown less malaria cases in screened houses than in controls [6]. Moreover, the association between the incidence of malaria and the accessibility of a population to mosquitoes was observed with the highest incidence in the population most accessible for mosquito bites [8]. In The Gambia, screening doors, windows and eves resulted in 59% reduction of indoors density of *An. gambiae,* and reduced the prevalence of anaemia [11]. Screening houses by plastic insect-screen resulted 80% protection from indoor bites of *An. gambiae* in The Gambia [9].

The likely explanation for moderate efficacy the current intervention is that people may not use screened doors in the nights before collection because the screened doors were not permanently fixed as windows. Moreover, *An. arabiensis* could enter houses when the people open the doors during earlier hours of the night [16]. The small gaps left in the door and windows could also contribute for the moderate reduction of mosquitoes in the intervention houses. Maximum reduction in number of *An. arabiensis* might be achieved if the screened doors were constantly used by home owner’s, and the doors were compatible for screening. The likely reason for the overall lower number of mosquitoes sampled during intervention (October/November 2011) compared to the pre-intervention period (April/May 2011) was presumably due to the seasonal variation of the area. Study from the same area shows the highest density of mosquitoes in April and May; the months with the highest rainfall than the October and November; the months with short and small rains [4,14]*.*

The intervention was cheap, and simple to implement and hence, it can be incorporated into an integrated vector management strategy, and combined with IRS and LLITNs. The cost for screening doors and windows and closing openings on eves and walls (7.3 USD per house) was lower than that was used for fully screening houses (9.98 USD per person) and for screening ceilings (8.69 USD per person) in The Gambia [11]. However, improving doors and windows fitness for screening should be considered during house construction to increase the efficacy of screenings.

## Competing interests

The authors declared that they have no competing interests.

## Authors’ contributions

FM: Project design, conducted field and laboratory work, data analysis and interpretation, wrote the draft manuscript, BL: Project design, field supervision, provided statistical input and manuscript revision. All authors read and approved the final manuscript.

## References

[B1] N’Guessan R Corbel V Akogbéto M Rowland M Reduced Efficacy of insecticide-treated nets and indoor residual spraying for malaria control in pyrethroid resistance area, Benin Emerg Infect Dis 2007 13 199 206 10.3201/eid1302.060631 17479880 PMC2725864

[B2] Balkew M Ibrahim M Koekemoer LL Brooke BD Engers H Aseffa A Gebre-Michael T Elhassen I Insecticide resistance in *Anopheles arabiensis* (Diptera: Culicidae) from villages in central, northern and south west Ethiopia and detection of kdr mutation Parasit Vectors 2010 3 40 10.1186/1756-3305-3-40 20416109 PMC2868498

[B3] Yewhalaw D Wassie F Steurbaut W Spanoghe P Van Bortel W Denis L Tessema DA Getachew Y Coosemans M Duchateau L Speybroeck N Multiple insecticide resistance: an impediment to insecticide-based malaria vector control program PLoS ONE 2011 6 e16066 10.1371/journal.pone.0016066 21264325 PMC3020220

[B4] Massebo F Balkew M Gebre-Michael T Lindtjorn B Blood meal origins and insecticide susceptibility of *Anopheles arabiensis* from Chano in South-West Ethiopia Parasit Vectors 2013 6 44 10.1186/1756-3305-6-44 23433306 PMC3606335

[B5] Beier J Keating J Githure JI Macdonald MB Impoinvil DE Novak RJ Integrated vector management for malaria control Malar J 2008 7 S4 10.1186/1475-2875-7-S1-S4 19091038 PMC2604879

[B6] Lindsay SW Emerson PM Charlwood JD Reducing malaria by mosquito-proofing houses Trends Parasitol 2002 18 510 514 10.1016/S1471-4922(02)02382-6 12473368

[B7] Fullerton H Bishop EL Improved rural housing as a factor in malaria control South Med J 1933 26 465 468 10.1097/00007611-193305000-00024

[B8] Boyd M The influence of obstacles unconsciously erected against anophelines (housing and screening) upon the incidence of malaria Am J Trop Med 1926 6 157 160

[B9] Lindsay S Jawara M Paine K Pinder M Walraven GE Emerson PM Changes in house design reduce exposure to malaria mosquitoes Trop Med Int Health 2003 8 512 517 10.1046/j.1365-3156.2003.01059.x 12791056

[B10] Lindsay S Snow RW The trouble with eaves; house entry by vectors of malaria Trans R Soc Trop Med Hyg 1988 82 645 646 10.1016/0035-9203(88)90546-9 3256125

[B11] Kirby M Ameh D Bottomley C Green C Jawara M Milligan PJ Snell PC Conway DJ Lindsay SW Effect of two different house screening interventions on exposure to malaria vectors and on anaemia in children in The Gambia: a randomised controlled trial Lancet 2009 374 998 1009 10.1016/S0140-6736(09)60871-0 19732949 PMC3776946

[B12] Kirby M Bah P Jones COH Kelly AH Jasseh M Lindsay SW Social acceptability and durability of two different house screening interventions against exposure to malaria vectors, *Plasmodium falciparum* infection, and anemia in children in The Gambia, west Africa Am J Trop Med Hyg 2010 83 965 972 10.4269/ajtmh.2010.10-0311 21036822 PMC2963954

[B13] Loha E Lindtjorn B Predictors of *Plasmodium falciparum* malaria incidence in Chano Mille, South Ethiopia: a longitudinal study Am J Trop Med Hyg 2012 87 450 459 10.4269/ajtmh.2012.12-0155 22826493 PMC3435347

[B14] Massebo F Balkew M Gebre-Michael T Lindtjørn B Entomologic inoculation rates of *Anopheles arabiensis* in South-western Ethiopia Am J Trop Med Hyg 2013 89 466 473 10.4269/ajtmh.12-0745 23878184 PMC3771283

[B15] Gillies M Coetzee M A supplement to the anopheline of Africa South of Sahara S Afr Inst Med Res 1987 55 143

[B16] Yohannes M Boelee E Early biting rhythm in the Afro-tropical vector of malaria, *Anopheles arabiensis*, and challenges for its control in Ethiopia Med Vet Entomol 2012 26 103 105 10.1111/j.1365-2915.2011.00955.x 21410494

